# The Mobile-Based 6-Minute Walk Test: Usability Study and Algorithm Development and Validation

**DOI:** 10.2196/13756

**Published:** 2020-01-03

**Authors:** Dario Salvi, Emma Poffley, Elizabeth Orchard, Lionel Tarassenko

**Affiliations:** 1 Institute of Biomedical Engineering Department of Engineering Science University of Oxford Oxford United Kingdom; 2 Department of Cardiology Oxford University NHS Foundation Trust Oxford United Kingdom

**Keywords:** cardiology, exercise test, pulmonary hypertension, mobile apps, digital signal processing, global positioning system

## Abstract

**Background:**

The 6-min walk test (6MWT) is a convenient method for assessing functional capacity in patients with cardiopulmonary conditions. It is usually performed in the context of a hospital clinic and thus requires the involvement of hospital staff and facilities, with their associated costs.

**Objective:**

This study aimed to develop a mobile phone–based system that allows patients to perform the 6MWT in the community.

**Methods:**

We developed 2 algorithms to compute the distance walked during a 6MWT using sensors embedded in a mobile phone. One algorithm makes use of the global positioning system to track the location of the phone when outdoors and hence computes the distance travelled. The other algorithm is meant to be used indoors and exploits the inertial sensors built into the phone to detect U-turns when patients walk back and forth along a corridor of fixed length. We included these algorithms in a mobile phone app, integrated with wireless pulse oximeters and a back-end server. We performed Bland-Altman analysis of the difference between the distances estimated by the phone and by a reference trundle wheel on 49 indoor tests and 30 outdoor tests, with 11 different mobile phones (both Apple iOS and Google Android operating systems). We also assessed usability aspects related to the app in a discussion group with patients and clinicians using a technology acceptance model to guide discussion.

**Results:**

The mean difference between the mobile phone-estimated distances and the reference values was −2.013 m (SD 7.84 m) for the indoor algorithm and −0.80 m (SD 18.56 m) for the outdoor algorithm. The absolute maximum difference was, in both cases, below the clinically significant threshold. A total of 2 pulmonary hypertension patients, 1 cardiologist, 2 physiologists, and 1 nurse took part in the discussion group, where issues arising from the use of the 6MWT in hospital were identified. The app was demonstrated to be usable, and the 2 patients were keen to use it in the long term.

**Conclusions:**

The system described in this paper allows patients to perform the 6MWT at a place of their convenience. In addition, the use of pulse oximetry allows more information to be generated about the patient’s health status and, possibly, be more relevant to the real-life impact of their condition. Preliminary assessment has shown that the developed 6MWT app is highly accurate and well accepted by its users. Further tests are needed to assess its clinical value.

## Introduction

### Background

The 6-min walk test (6MWT) is a common clinical instrument for assessing patients’ functional capacity. It consists of instructing patients to walk as far as they can during 6 min, usually in a corridor [1], under the observation of a doctor or a physiologist. The primary measurement of the test is the total distance walked, computed as the total number of lengths or laps walked plus the excess distance measured with a trundle wheel, a measuring tape, or with marks along the corridor. Secondary measures can include fatigue and dyspnea, measured with a modified Borg or analogue scale and peripheral arterial oxygen saturation via pulse oximetry. The 6MWT is self-paced, and patients are unlikely to push themselves beyond their endurance or through musculoskeletal pain. The test is easy to administer, well tolerated, and reflects activities of daily living better than other walk tests [2].

The walked distance reflects exercise capacity determined by maximal cardiopulmonary exercise testing in patients with cardiopulmonary conditions and has a strong association with mortality in primary pulmonary hypertension [3], heart failure [4], and chronic obstructive pulmonary disease (COPD) [5]. The test is also used for assessing the effect of therapies such as pulmonary rehabilitation, oxygen therapy, long‐term use of inhaled corticosteroids, and lung volume reduction surgery [6]. In the interpretation of the results, a change in walking distance of more than 50 m is usually considered clinically significant in most disease states [6].

Although the test is easy to perform, it involves costs and some practical limitations. To start with, it requires a dedicated corridor in the hospital, of length between 30 m and 50 m and no shorter than 15 m [7]. It also requires a physiologist to observe the test and to note down the measurements. Patients need to get to the hospital clinic where the test is performed, sometimes from a long distance, with associated costs of transport and the accompanying stress for the patient. Owing to these limitations, the 6MWT cannot be performed very often. For example, pulmonary hypertension patients are invited to perform a test every 3 to 6 months only [8].

With the advent of affordable digital devices and mobile phones, it becomes possible to perform the test in (or near) the patient’s home, using sensors such as accelerometers or the global positioning system (GPS) to estimate the distance walked.

### Objectives

In this paper, we present a mobile phone app which enables patients to perform the 6MWT on their own, at their convenience or in the hospital setting, while augmenting the information collected during the test using off-the-shelf portable pulse oximeters.

### Related Work

The walked distance can be obtained using satellite positioning systems when outdoors and with inertial sensors when indoor.

Positioning systems like GPS are already widely used for estimating distance in the automotive sector. Modern GPS receivers provide a signal which is the result of heavy processing and is usually improved and smoothed with well-known techniques [9]. When used with human beings, these systems are known to introduce some error because of the inherent noise that exists in the GPS system and the lower distances travelled [10-12]. Nonetheless, the error is such that it has been considered negligible in previous work when GPS has been applied to estimate distance walked in the 6MWT [13]. As most algorithms used by GPS devices are proprietary, there is a lack of the literature describing how to derive the distance from the raw positions, except for the obvious computation of the distance between the first and the last received positions [12].

With regard to the indoor scenario, there is rich literature related to gait analysis with accelerometers [14-17]. From gait analysis, it is possible to compute the number of steps, which, once multiplied by the length of the step, would provide the distance walked.

In a study by Schimpl et al [18], 12 different algorithms for extracting human speed (and thus distance walked) from accelerometer data were explored. Some proposed algorithms make use of the step length as an input, whereas others rely purely on the accelerometry. From a validation against data recordings obtained from 17 subjects walking at different speeds, the authors found that the best performing algorithm was a support vector regression algorithm that was previously trained on an independent dataset recorded from 15 subjects who participated in 3 outdoor data collection activities. A similar approach was also followed in a study by Cheng et al [19] but using a mobile phone instead of a dedicated sensor. Data were processed in both the time and frequency domains, and 8 gait parameters were extracted as the inputs to a support vector regression model to estimate gait speed. The approach was validated with 6 COPD patients and 6 healthy subjects performing a 6MWT. These machine learning approaches, although accurate, rely heavily on the training data, and may be biased toward the walking style adopted during the data acquisition or the actual devices used.

Gait analysis–based approaches have also been used for the 6MWT. For example, in a study by Schulte et al [20], a telemonitoring system for 6MWT based on body-worn accelerometers was proposed. A simple step-detection algorithm was employed and combined with patient height data to estimate the distance walked. A more sophisticated approach was taken by Capela et al [21,22]; they used a mobile phone app to count steps and identify when the user turns while walking back and forth along a corridor. As the distance walked between U-turns is fixed, it is possible to estimate the step length and, thus, the residual distance walked after the last U-turn by multiplying the number of steps by the stride length. The algorithm uses the azimuth signal provided by Blackberry phones, which is, in turn, estimated from the gyroscope and the magnetometer sensors. Some corrections are introduced to this signal to smooth sudden variations, for example, detecting a turn if the signal changes by more than 100° in 3 seconds. The approach was validated with 15 volunteers and led to less than 1 m average error.

In addition to research papers, it is also worth mentioning the Apple Research Kit, an open-source software framework that allows developers to build mobile health (mHealth) apps with a set of already implemented use-cases. One of these use-cases is the *timed walk*, which can be used to implement the 6MWT. The timed walk activity estimation has already been used in a few studies [23,24], even though the accuracy of the algorithm used to estimate the distance, based on the Core Motion framework, is not publicly disclosed. In a recent study [25], after having used Core Motion for 6MWT with peripheral artery disease (PAD) patients, authors concluded that “the iPhone’s built-in distance algorithm is unable to accurately measure distance, suggesting that custom algorithms are necessary for using iPhones as a platform for monitoring distance walked in PAD patients.”

## Methods

### System Design

Our system was co-designed by a team of engineers, cardiologists, physiologists, and patients in a set of *discussion groups* [26], which were part of our Patient and Public Involvement in research strategy [27]. The team focused mainly on patients with pulmonary hypertension, especially because they are treated with pulmonary vasodilator therapies, which are expensive, and uptitration of these depends partly on the results of the 6MWT [28]. There are many types of pulmonary hypertension including idiopathic, chronic thromboembolic, secondary to congenital heart disease, secondary to respiratory, or cardiac disease [29], and therefore, the demographics of the patients can be diverse, for example, patients with congenital heart disease have different characteristics compared with patients with interstitial lung disease. The research team decided to recruit adults, without learning difficulties, who were familiar with mobile phones. Patients had to be able to walk independently and were not using oxygen therapy.

It was also decided to make use of patients’ own phones, instead of providing them with dedicated ones. This was because we hypothesized that users would prefer using the devices with which they are familiar; however, this meant that both Android and iPhones had to be supported. This decision also allowed us to collect information about free-living physical activity, as gathered by the phone’s sensors or any wearable connected to it.

Given that these patients can desaturate significantly during exertion, it was decided to acquire pulse oximetry data during the 6MWT with a wireless sensor attached to the patient’s finger. To complement the observations made by the physiologist with these data, the mobile phone also had to be used during the test at the hospital. In addition, the clinician responsible for the patient’s care, in this case the cardiologist, had to be given an interface to review all the patient’s data collected by the system.

To summarize these requirements, the following use-cases were identified:

1. A patient performs the 6MWT in the hospital, while being monitored by a mobile phone app.
2. A physiologist supervising the 6MWT enters the observed outcomes on a tablet computer.
3. A patient performs the 6MWT outdoors, in a place of their choice.
4. A patient sends their physical activity data, as measured by passive monitors and activity trackers over the duration of a week.
5. A clinician reviews patient’s data on a website.

**Figure 1:**
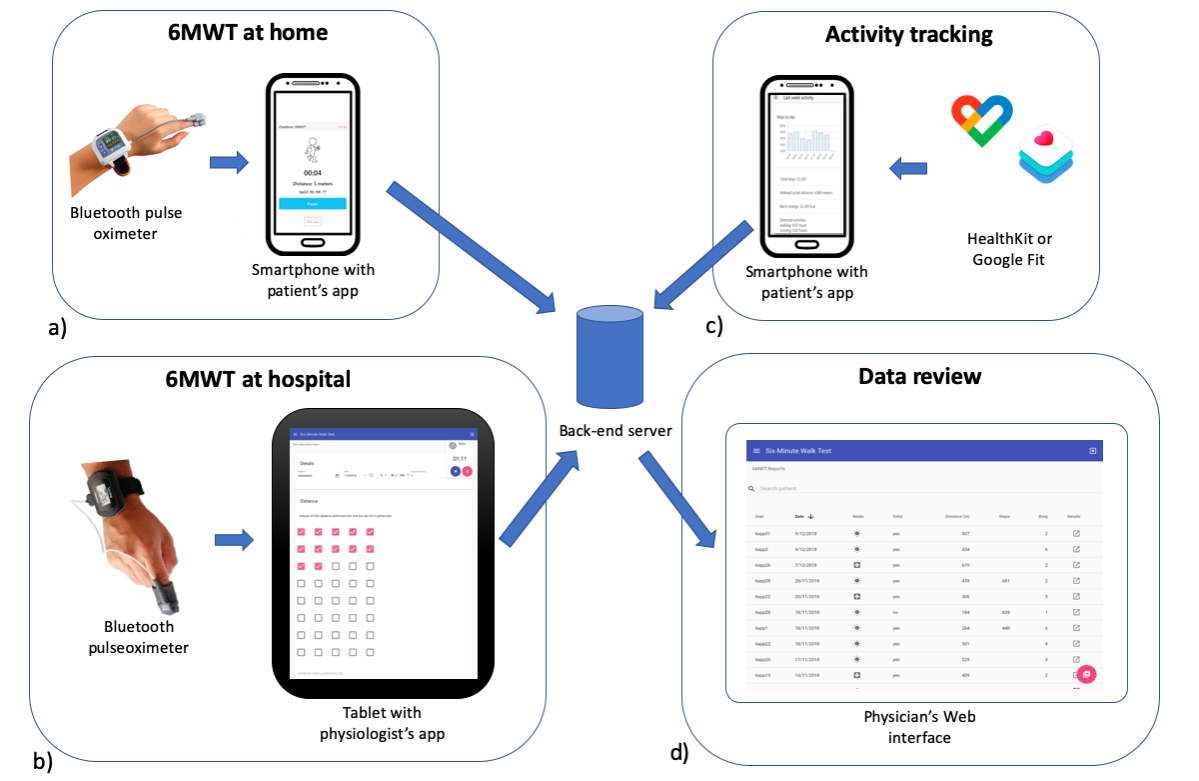
Architecture of the 6-min walk test (6MWT) system. It includes 4 scenarios: (a) 6MWT at home, where patients perform a 6MWT in their home setting using their mobile phone with the app and a wireless pulse oximeter; (b) 6MWT in the hospital, where patients perform the test while being observed by a physician and with pulse oximetry data being collected through a tablet app; (c) activity tracking data retrieved by Google Fit or HealthKit transmitted for subsequent analysis; (d) data review performed by a physician through a Web interface.

To support the abovementioned use-cases, we designed the client-server architecture shown in Figure 1.

The server includes a database and a website to collect patient data to be subsequently reviewed by clinicians. Physiologists can use a tablet computer with an app that allows them to review patients’ information and report the results of the 6MWT. The app also allows connection to a wireless pulse oximeter to retrieve peripheral arterial oxygen saturation (SpO_2_) and heart rate values, while the patient is performing the test.

Patients are provided with a mobile phone app, downloaded onto their phones, which allows both indoor and outdoor 6MWT. The indoor test is performed on a walkway of a known length, for example, in a hospital clinic. The outdoor test can be performed in any place where there is a GPS signal of sufficient strength. At the end of each test, the data are sent to the server to be reviewed by clinicians. Patients can also send data about passive activity monitoring using HealthKit for iOS and Google Fit for Android. These can compute steps and activity through either mobile phone sensors or other connected apps.

Patient data are protected by means of well-established techniques, that is, users are authenticated with a username and password, and data are transmitted over an http encrypted channel.

### Distance Estimation

To compute the distance walked, we developed and tested 2 algorithms: one for the indoor scenario and one for the outdoor scenario.

The accuracy of the algorithms was estimated by performing a set of indoor and outdoor 6MWTs, with the app running on a mobile phone held in one hand and a trundle wheel held in the other hand. Different types of walking styles (from slow to fast), path curviness (from straight to U-turns), and hand shakiness (from still to slightly shaky) were simulated.

Most tests were executed by researchers in lab settings; however, to fine tune the indoor algorithm in real-life conditions, we also asked some patients to hold the mobile phone while performing the 6MWT during a regular clinic visit. Only the distance and an approximate age range were collected.

Accuracy was calculated using the mean, median, and standard deviation of the difference between the reference values and the outputs from our algorithm; the mean, standard deviation, minimum and maximum of the absolute difference; and the Pearson correlation between estimated values and the reference values. In addition, Bland-Altman plots were also generated.

Details about the algorithms are provided as follows.

### Indoor Distance Estimation Algorithm

We tried to implement the algorithm described by Capela et al [22], but, possibly as we employed a different operating system, or possibly because of the lack of details in the paper, our implementation was not capable of detecting any U-turn. We therefore decided to develop a new approach.

**Figure 2:**
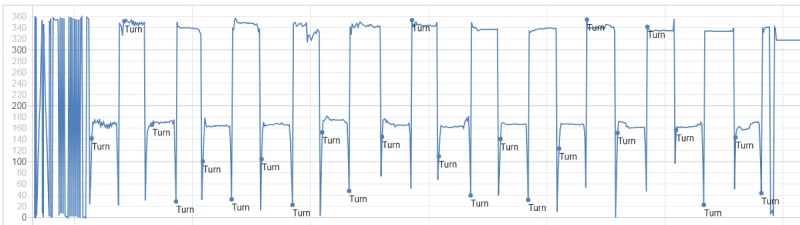
Example of mobile phone azimuth signal. The first seconds show the calibration phase, after which U-turns are detected when the difference between near angles becomes greater than the set threshold within a short time window.

The underlying concept is to detect changes of 180° in the mobile phone azimuth signal (an example of such a signal is shown is Figure 2). To this end, we made use of the *virtual compass* provided by the Android and iOS programming interfaces, which computes the azimuth by combining the signals provided by the accelerometer and the magnetometer. The result of this estimation may lead to some distortions and inaccuracies but, given that we are only interested in large *changes* in the azimuth signal, these are mostly negligible. One exception is the fact that the maximum amplitude of the signal, on which the algorithm relies, may be less than 360°. To account for this, the algorithm requires a simple calibration phase, in which users are asked to execute a 360° turn with the mobile phone in one hand, so that the minimum and maximum azimuths are captured.

After calibration, samples of the compass signal are acquired every 500 ms. If the current sample is more than *min_turn_time* milliseconds away from either the start of the test or the latest detected U-turn, the sample is compared against past samples collected in a buffer. If the minimum difference between the angles of any of those samples is less than a predetermined threshold, then a U-turn is detected.

The parameters to be optimized in this algorithm are *min_turn_time*, the length of the buffer, and the threshold, which is set to be 35% of the difference between the maximum and the minimum values observed during the calibration. As for the *min_turn_time*, it is given an initial value of 10 seconds, and then adapted to the actual walking speed of the patient; each time a U-turn is detected, *min_turn_time* is updated to be the minimum observed time needed to complete a lap, minus 20%. If *min_turn_time* is such that the speed of the patient would be more than 2 m/s, it is capped. The length of the buffer is initialized to contain 4 seconds’ worth of samples. This number of seconds is then updated, every time a U-turn is detected, to half of *min_turn_time*, but capped at 5 seconds.

Once U-turns are detected, the walked distance is computed by multiplying the number of U-turns by the length of the lap. Any residual time between the last U-turn and the end of the test is accounted for by multiplying it by the median of the detected times between U-turns.

If a step counter is available, it is used to improve the residual distance estimation. Specifically, instead of using the median *between U-turns* completion time, the average step length (ie, the total distance divided by the total number of steps up to the last U-turn) is multiplied by the residual number of steps since the last U-turn.

The source code of the algorithm is provided in the Multimedia Appendix 1.

### Outdoor Distance Estimation Algorithm

**Figure 3:**
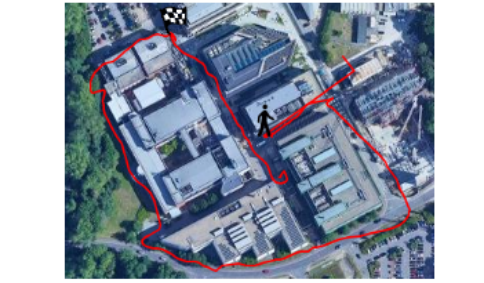
Example of a positioning trace (in red) retrieved from the mobile phone. The walking man figure indicates the starting point of the test; the flag indicates its end. Comparing the trace with the underlying picture shows that the position is sometimes affected by an error, for example, near tall buildings which reflect the signal or because of trees obscuring the global positioning system satellite’s signal.

The outdoor algorithm works by using the localization information provided by the GPS system embedded in the phone (an example is shown in Figure 3). The principle is based on down-sampling the positioning signal, calculating the *as the crow flies* distance between each sample and summing up the distances thus obtained. Down-sampling is used as a strategy to reduce the noise contained in the signal, but instead of simply taking a sample every x seconds, we adopted a more sophisticated approach.

As the mobile phone GPS system needs some time to be fully connected, we let the user wait until a *good* signal is available. Both Android and iOS operating systems provide an error estimation for each positioning sample, based on the number of visible satellites. We experimentally observed in lab tests that an error lower than 15 m indicates that the GPS system has found enough satellites to localize the phone and, likely, the error would decrease further. If the error does not decrease below 15 m within 2 min after the start, the test is flagged as possibly affected by low accuracy.

After this simple signal quality step, positioning samples start to be collected. Every *selection_period* seconds, the algorithm selects the sample with the lowest estimated error within those available within the last 25% of *selection_period*. Once a sample has been selected, the distance between the previous selected sample and the newly selected one is computed and added to the total.

**Figure 4:**
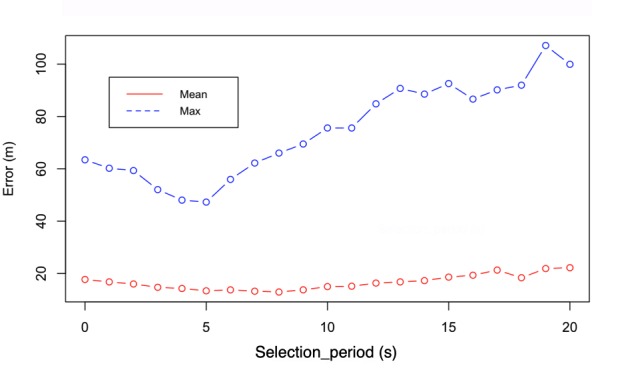
Maximum and mean error of the distance estimation versus the sampling period of the localization signal selection_period computed on all available tests. The 5 seconds value minimizes both mean and maximum error.

The *selection_period* parameter needs to be optimized. A faster sampling period will allow more samples to be used and therefore more noise to be accounted for in the distance estimation. A longer period is problematic if the path walked by the user is not straight (in a perfectly straight path, in fact, just the first and the last sample would suffice). To optimize this parameter, we computed the mean and maximum distance estimation error using the tracks acquired during our tests and varying *selection_period* from 0 second to 20 seconds with 1- seconds steps. The value that minimizes both mean and maximum error is 5 seconds, as shown in Figure 4.

If step counting is available, we use it to exclude samples for which the number of steps does not increase. For example, a perfectly still mobile phone will produce positioning samples with jitter around them because of noise, and the algorithm will sum up the distances between them. By using the step counter, it is possible to identify when the user is still, thus not accumulating those erroneous distances.

The source code of the algorithm is provided in the Multimedia Appendix 2.

### User Aspects

To understand user aspects such as the usability and technology acceptance of our system, we organized a discussion group to collaboratively analyze one of the first prototypes that had been developed. Different types of stakeholders were invited to the group including patients, physiologists, physicians, and engineers. Participants were informed that the outcomes of the discussion could be used for scientific publications, and patients were required to sign an informed consent form.

To structure the content of the discussion group, we used the mHealth technology acceptance model form [30], which integrates common technology acceptance and health behavior theories. The model includes 8 constructs: response efficacy, perceived ease of use, subjective norm, response cost, self-efficacy, perceived vulnerability, perceived severity, and intention to adopt. Of these, we selected perceived ease of use, response cost, self-efficacy, response efficacy, perceived vulnerability, and intention to adopt as the most appropriate to our project and stage of development.

The discussion group was led by one researcher, who explained the system and its capabilities and asked the attendees questions. The content was split into 5 parts. In the first part, 2 questions were asked to understand what the current limitations and needs were in relation to a conventional 6MWT:

- Q1. What are the most annoying things about the 6MWT as it is done now?
- Q2. How does the test compare with your normal level of fitness; do the test’s results adequately reflect the way you feel?

After a discussion of the answers to these 2 questions, a general presentation of the system was given (part 2), after which, other questions were asked about the overall concept (part 3):

- Q3. What do you think are the advantages of the system you have just seen?
- Q4. What are the disadvantages?

The fourth part of the discussion consisted in letting patients download the app on their phone and use it in a test run, while the research team recorded difficulties, technical issues, and general comments.

Finally, in part 5, a further set of questions were asked about usability and acceptance:

- Q5. Do you find the system easy to use?
- Q6. Would you suggest any changes to it?
- Q7. Do you see yourself performing the test at home?
- Q8. Would you need someone to help you?
- Q9. Would you use the indoor or the outdoor test?
- Q10. How often do you think you will be doing it?
- Q11. Do you see yourself using the app in the long term (2 years or more)?

These questions were mapped to the constructs under analysis as follows: perceived ease of use: Q5, self-efficacy: Q7 and Q8, response cost: Q4, response efficacy: Q3, perceived vulnerability: Q1 and Q2, intention to adopt: Q9, Q10, and Q11.

The discussion was audio-recorded for later analysis.

## Results

### System Design

We developed 2 apps, 1 for the patient and 1 for the physiologist, as well as a server for the back-end system.

Having decided to use the patients’ own mobile phones, we needed to support both Android and iOS operating systems. We therefore implemented the patients’ app using the Apache Cordova framework. In addition to Cordova, the app makes use of the Ionic framework (first version), which uses Angular as the front-end JavaScript framework.

To retrieve the data about passive monitoring, we connected the app to Google Fit on Android and HealthKit on iOS. Both systems provide an *aggregator* for wearables and fitness apps and are able to compute steps and basic activity recognition (still, walking, running, and on a vehicle) relying on the mobile phone’s sensors.

For ambulatory pulse oximetry, we chose the Nonin WristOx (only compatible with Android) and Creative Medical PC68B (compatible with both Android and iOS) because they are wrist-worn, with a finger probe, and because of their Bluetooth wireless connectivity.

**Figure 5:**
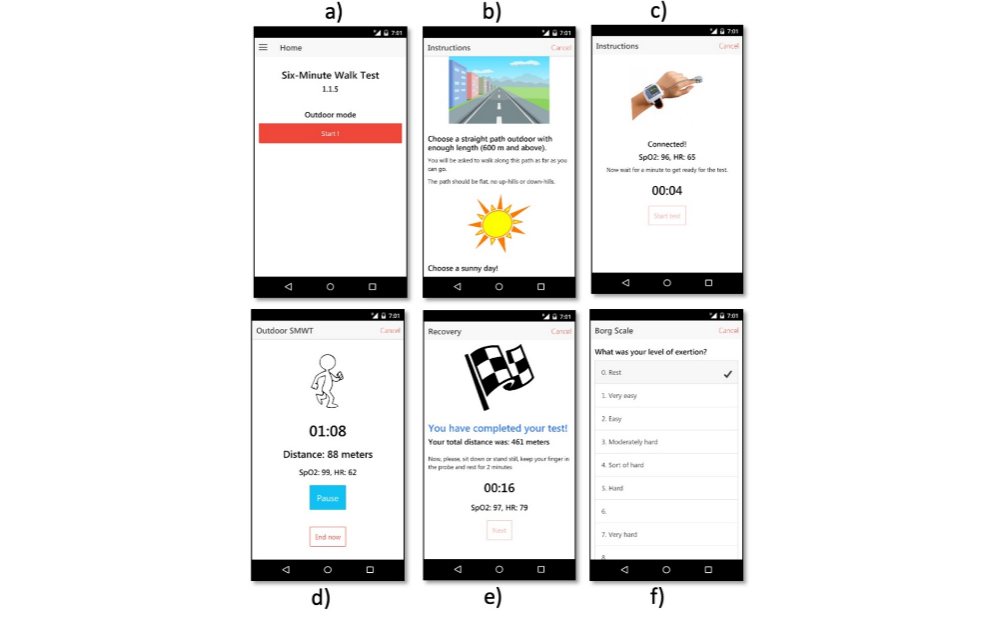
Screenshots of the patients’ app. (a) Home page, (b) instructions about how to perform the test, (c) connection to the pulse oximeter and baseline measurements at rest, (d) estimation of the distance during walk, (e) total distance estimation and recovery at rest, (f) Borg scale questionnaire.

The outdoor 6MWT use-case is shown in Figure 5: the patient triggers a new test on the mobile phone’s home screen; the app shows suggestions on how to perform the test in the best conditions, then waits for the pulse oximeter to be connected, and records a baseline measurement of heart rate and oxygen saturation at rest. The patient is then invited to walk for 6 min, during which the walked distance estimation and a timer are shown. The patient is allowed to pause or cancel the test at any point. At the end of the 6-min period, the patient is again invited to rest, while recovery heart rate and oxygen saturation are measured. Finally, the patient answers the Borg scale question and can add some general comments. During the test, the app also retrieves the local weather information from a Web-based service. This is used to explore the relationship between compliance or exertion levels and weather.

**Figure 6:**
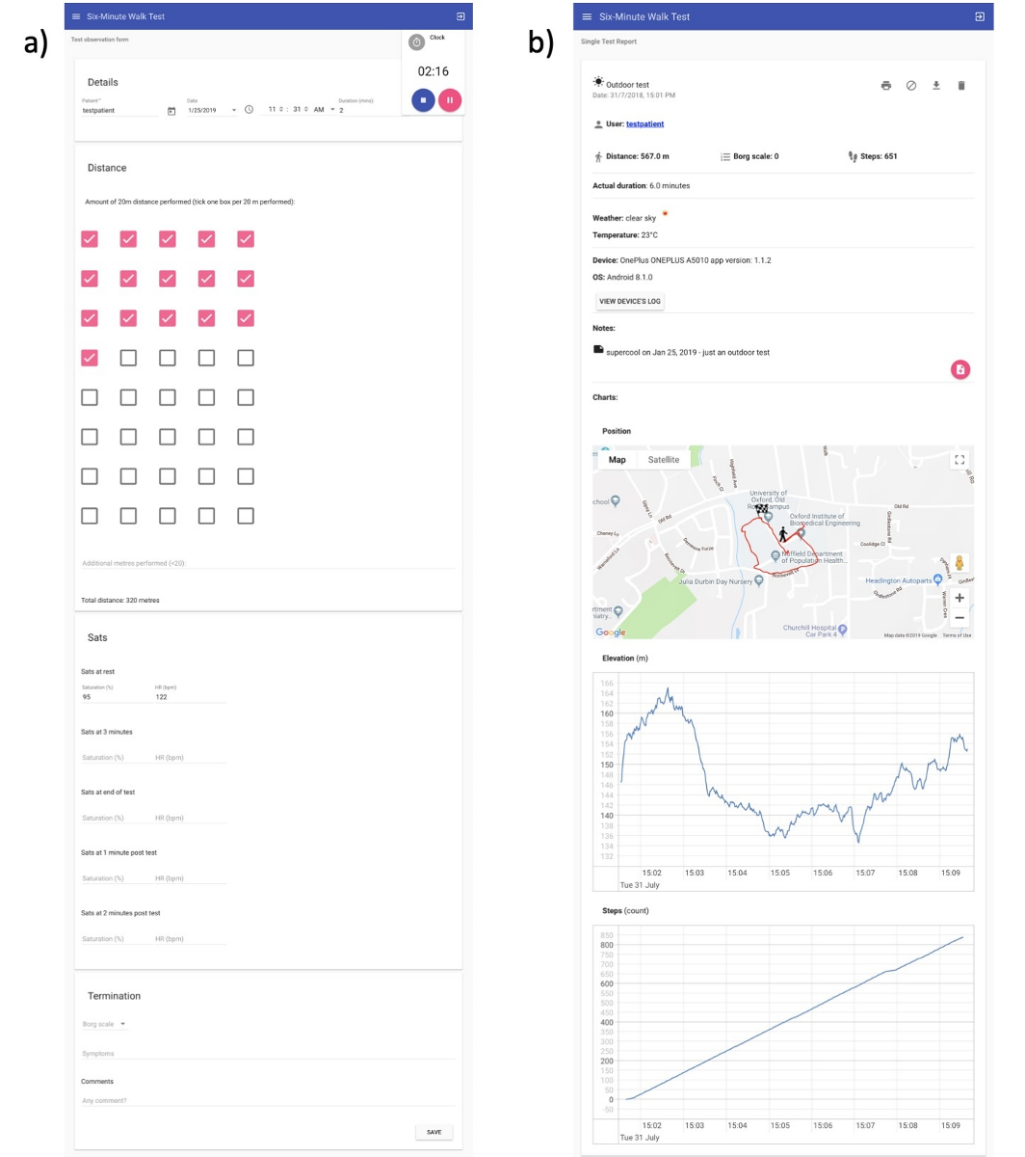
Screenshots of the server Web interface. (a) The form physiologists fill in when observing a 6-min walk test (6MWT), (b) an example of an outdoor 6MWT results (heart rate and oxygen saturation charts are omitted).

The server system consists of a nonrelational database (ArangoDB), a REST API developed with Nodejs, and a front-end website developed with Angular. The Web interface (Figure 6) allows physiologists and doctors to manage users, enter the results of a hospital 6MWT, review the data produced both by the app and hospital tests, and analyze trends for each patient. The physiologists’ app is a Cordova app that uses the same front-end code as used for the server with some modifications to allow the data to be retrieved from wireless pulse oximeters.

### Distance Estimation

A total of 79 indoor and outdoor tests were performed. Lab tests were undertaken by researchers, all males, aged 30, 33, and 37. The distance estimated in regular 6MWT clinics was collected 18 times from both male and female volunteers and with an age span of 15 to 85 years.

The accuracy of the algorithm is reported separately for the indoor and outdoor scenarios.

### Accuracy of the Indoor Algorithm

The accuracy of the indoor algorithm was estimated using results from 49 tests. The characteristics of the tests are shown in Table 1.

**Figure 7:**
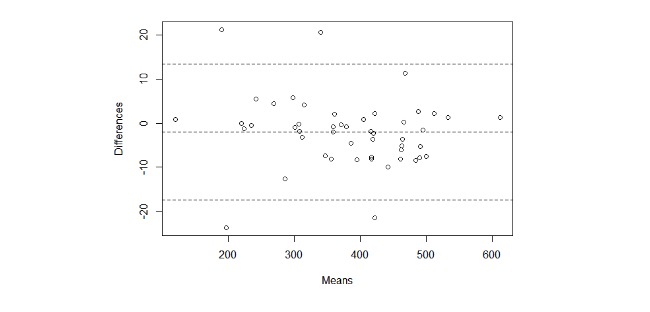
Bland-Altman plot of the difference between the estimated distance walked and the absolute distance. The Shapiro-Wilk test confirms the normality of the data (0.91).

The difference between the algorithm’s estimates and the measurements taken from the trundle wheel are summarized in Table 2, with the Bland-Altman plot shown in Figure 7.

**Table 1:** Summary characteristics of the indoor tests.

Characteristics	Value
Number of tests	49
Number of different phones tested	11
Walked distance measured by trundle wheel (m), mean (SD)	381.79 (103.90)
Steps, as estimated by the phone’s pedometer, mean (SD)	574.10 (146.30)

**Table 2:** Accuracy metrics for the indoor algorithm. By difference, we mean the difference between the estimated distance as computed by the app and the reference distance, as measured by the trundle wheel.

Accuracy metric	Value
Mean difference (m)	−2.01
Median difference (m)	−1.51
Standard deviation of the difference (m)	7.84
Correlation	0.99
Mean absolute difference (m)	5.55
Standard deviation of the absolute difference (m)	5.84
Minimum absolute difference (m)	0
Maximum absolute difference (m)	23.68

### Accuracy of the Outdoor Algorithm

The characteristics of the outdoor tests are shown in Table 3.

**Figure 8:**
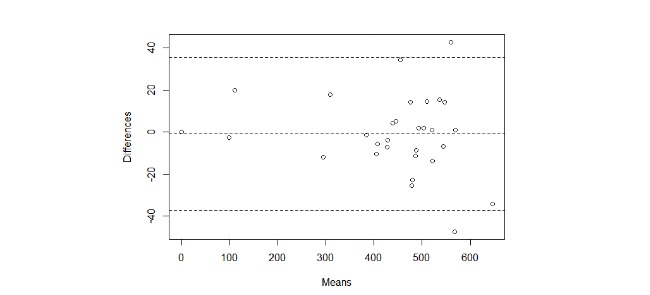
Bland-Altman plot of the difference between the estimated distance walked and the ground truth. The Shapiro-Wilk test confirms the normality of the data (0.97).

The accuracy metrics are listed in Table 4, with the Bland-Altman plot shown in Figure 8.

**Table 3:** Characteristics of the outdoor tests.

Characteristic	Value
Number of tests	30
Number of different phones tested	8
Walked distance measured by trundle wheel (m), mean (SD)	437.99 (147.82)
Steps, as estimated by the phone’s pedometer, mean (SD)	696.5 (78.31)

**Table 4:** Accuracy metrics of the outdoor algorithm. By difference, we mean the difference between the estimated distance as computed by the app and the reference distance, as measured by the trundle wheel.

Accuracy metric	Value
Mean difference (m)	−0.80
Median difference (m)	−0.63
Standard deviation of the difference (m)	18.56
Correlation	0.99
Mean absolute difference (m)	13.39
Standard deviation of the absolute difference (m)	12.65
Minimum absolute difference (m)	0
Maximum absolute difference (m)	47.27

### User Aspects

The discussion group mentioned in the Methods section was held shortly after the first version of the app was ready. The attendees were 2 engineers, 1 cardiologist, 1 nurse, 2 physiologists, and 2 patients with pulmonary hypertension. One engineer led the discussion, while the other took notes.

From the initial discussion, the main issues with the way that the 6MWT is currently performed in hospital were identified as follows:

1. The corridor being used in the hospital was not ideal, as it was usually busy with other people and patients being moved on trolleys, both of which may affect the walking pace.
2. Patients’ performance might depend on their health status on that particular day and may not reflect their average status.
3. The test is only performed rarely (once or twice a year), and episodes of health deterioration may be missed.
4. Younger patients might underperform as opposed to the older ones who may try harder in the hospital test, which might not reflect real-life conditions.
5. White coat syndrome may cause anxiety in some patients and affect their performance.
6. Overall, patients are stressed and rushed in hospital.

After the app and monitoring system were presented, the following advantages were identified:

1. The system allows the patient to perform the test in a more comfortable environment and more often than the hospital tests.
2. Patients can see for themselves how they are progressing.
3. The system can alert in the case of very low oxygen saturations.

In terms of disadvantages:

1. When the test is performed outdoors, the weather can affect the patient’s performance.
2. Changes in altitude, as a result of walking up an incline, can affect the results of the test.

During the dry-run test of the system with 2 patients, the following observations were made:

1. The pulse oximeter generates a sound when a low oxygen saturation value is measured, but this can be disabled.
2. A patient had problems understanding how to wear the pulse oximeter.
3. Patients were able to install the app correctly and could understand its structure easily.
4. One patient tried to send their activity tracking data but did not have Google Fit installed.
5. Patients struggled to log in, because complex passwords with capital and lower case letters were originally assigned to them.
6. During a test, the pulse oximeter produced artefactual values.
7. A physiologist asked to be able to discard the results of a test if the data recorded did not appear to be accurate enough to them.

With respect to the usability of the app, the patients’ comments were as follows:

1. The app is usable and easy to understand.
2. It would be better to show how many seconds are left until the end of the test, rather than how many seconds have elapsed since the start.
3. One patient asked for a sound to be generated at the end of the test to allow them not to have to look at the mobile phone screen all the time.

Regarding the willingness to use the app, the following points were noted:

1. Both patients said that they would use the app regularly and would not need any help to do so.
2. One patient would prefer performing the test indoors during winter because the cold weather affects their breathing, whereas the other patient only wanted to use the outdoor version.
3. As indoor tests require a long passageway, it was suggested that shopping malls could be used for these tests.
4. One patient would like to place the mobile phone on an armband or in a pocket during the test.
5. Patients identified scenarios for which they could perform the test while doing some other activity, for example, taking children to and from school.
6. Both patients agreed to use the app in the long term.

## Discussion

### Distance Estimation

The results from our tests show that, in both the indoor and outdoor scenarios, the difference between the distance estimated by the app and the ground truth is always below 54 m, when 50 m is considered to be the clinically significant threshold for detecting changes in disease state [6].

If we compare our accuracy results with those reported in the literature, for the outdoor scenario, an average error of a few meters, up to a maximum of 20 m, was reported by Gray et al [10], which is consistent with our findings, although our maximum difference was higher. For the indoor scenario, in a study by Schulte et al [20], an average absolute error of 4% was reported, as compared with our 0.02%. One difference between the approach adopted by Capela et al [21] and ours is that the authors used inertial sensors worn on the body, not the sensors in a mobile phone. In the studies by Capela et al [21,22], the reported maximum error in distance estimation was 2 m compared with our maximum of 23 m. We should point out, however, that the method described in those papers was only validated with 1 phone and with 15 tests, as compared with our 11 mobile phones and 49 tests.

As limitations to our approach, we should mention that most of the accuracy tests were performed by researchers in a lab environment. Although we tried to simulate worst-case scenarios, it is possible that the distance estimation algorithm may perform worse *in the wild*. For example, the indoor algorithm is based on the assumption that the azimuth signal is not very noisy, and it requires the user to hold the phone relatively still, which may be not always be the case for some users. The outdoor algorithm is instead based on the assumption that the path walked by the user is straight or gently curved. To mitigate these factors, the app displays clear instructions before each test is initiated. In addition, we are planning to deliver a leaflet with written instructions and to provide a short training session before regular use of the app by the patient.

### User Aspects

In terms of user aspects, the results from the discussion group suggest that the way the 6MWT is performed in hospitals has significant limitations (a *perceived vulnerability* [30]) that our system, at least partially, addresses, thus offering *response efficacy* in relation to that vulnerability. But a *response cost*, that is, limitations to the validity of the measurements in the case of bad weather or a slope, was also pointed out.

In terms of *perceived ease of use*, patients were immediately able to use the app by themselves. This may also be justified by the fact that they regularly used a mobile phone and, thus, were also highly *self-efficient*.

Given the positive answers provided for its associated constructs, *intention to adopt* would be expected to be high. Indeed, patients declared to be willing to try the app. The fact that they tried to contextualize its use in their life, for example, while shopping or taking children to school, may indicate a genuine interest in this technology.

Although the indications of the discussion group were generally supportive of the system, there are risks that can affect its actual use; particularly, the fact that it has to be either used outdoors, which is limited by the weather, or indoors but in a long corridor, which is limited by the availability of space. In addition, the integration with the sensor and external apps makes the overall user experience more cumbersome, a fact that may affect some less tech-savvy users.

### Conclusions and Future Work

The system described in this paper allows patients to perform the 6MWT at a place of their convenience, thus allowing more information to be generated about their general health status, more frequently, and possibly reflecting their general health status better. The algorithms implemented to estimate the distance walked from either the compass or GPS showed good agreement with the reference trundle wheel measurements, with errors below the clinically significant threshold. Our preliminary user validation also indicates that the app is usable and has the potential to be well accepted among patients.

The system will need to be tested with more patients to assess its feasibility in a real-world scenario. A clinical trial is currently being run with 30 pulmonary hypertension patients to understand the relationship between tests undertaken using the app and conventional in-hospital tests. Further studies will then be needed to assess the clinical significance of the tests in the community and the relationship between passive activity monitoring, for example, through wearables and in-hospital 6MWT results.

## Appendix

Multimedia Appendix 1 Javascript implementation of the indoor distance estimation algorithm.

Multimedia Appendix 2 Javascript implementation of the outdoor distance estimation algorithm.

## Abbreviations

6MWT: 6-min walk test
COPD: chronic obstructive pulmonary disease
GPS: global positioning system
mHealth: mobile health
PAD: peripheral artery disease
